# A Method for Quantifying Back Flexion/Extension from Three Inertial Measurement Units Mounted on a Horse’s Withers, Thoracolumbar Region, and Pelvis

**DOI:** 10.3390/s23249625

**Published:** 2023-12-05

**Authors:** Chloé Hatrisse, Claire Macaire, Camille Hebert, Sandrine Hanne-Poujade, Emeline De Azevedo, Fabrice Audigié, Khalil Ben Mansour, Frederic Marin, Pauline Martin, Neila Mezghani, Henry Chateau, Laurence Chèze

**Affiliations:** 1 Laboratoire de Biomécanique et Mécanique des Chocs (LBMC) UMR_T 9406, Université Gustave Eiffel, Université Claude Bernard Lyon 1, 69622 Lyon, France; laurence.cheze@univ-lyon1.fr; 2 CIRALE, USC 957 BPLC, Ecole Nationale Vétérinaire d’Alfort, 94700 Maisons-Alfort, France; claire.macaire@vet-alfort.fr (C.M.); emeline.de-azevedo@vet-alfort.fr (E.D.A.); fabrice.audigie@vet-alfort.fr (F.A.); henry.chateau@vet-alfort.fr (H.C.); 3 Laboratoire d’Innovation Ouverte en Technologies de la Santé (LIO), Centre de Recherche du Centre Hospitalier de l’Université de Montréal (CRCHUM), Montréal, QC H2X 0A9, Canada; neila.mezghani@teluq.ca; 4 Laboratoire de BioMécanique et BioIngénierie (UMR CNRS 7338), Centre of Excellence for Human and Animal Movement Biomechanics (CoEMoB), Université de Technologie de Compiègne (UTC), Alliance Sorbonne Université, 60200 Compiègne, France; khalil.ben-mansour@utc.fr (K.B.M.); frederic.marin@utc.fr (F.M.); 5 Labcom LIM-ENVA, LIM France, 24300 Nontron, France; chebert@lim-group.com (C.H.); shannepoujade@lim-group.com (S.H.-P.); pmartin@lim-group.com (P.M.)

**Keywords:** horse, back, thoracolumbar, flexion/extension, IMU

## Abstract

Back mobility is a criterion of well-being in a horse. Veterinarians visually assess the mobility of a horse’s back during a locomotor examination. Quantifying it with on-board technology could be a major breakthrough to help them. The aim of this study was to evaluate the accuracy of a method of quantifying the back mobility of horses from inertial measurement units (IMUs) compared to motion capture (MOCAP) as a gold standard. Reflective markers and IMUs were positioned on the withers, eighteenth thoracic vertebra, and pelvis of four sound horses. The horses performed a walk and trot in straight lines and performed a gallop in circles on a soft surface. The developed method, based on the three IMUs, consists of calculating the flexion/extension angle of the thoracolumbar region. The IMU method showed a mean bias of 0.8° (±1.5°) (mean (±SD)) and 0.8° (±1.4°), respectively, for the flexion and extension movements, all gaits combined, compared to the MOCAP method. The results of this study suggest that the developed method has a similar accuracy to that of MOCAP, opening up possibilities for easy measurements under field conditions. Future studies will need to examine the correlations between these biomechanical measures and clinicians’ visual assessment of back mobility defects.

## 1. Introduction

The horse’s back is a complex anatomical region occupying an essential place in the musculoskeletal system [1]. This region is highly stressed during sports and racing activities, particularly when it must support the rider’s weight [2]. Knowledge of the anatomy and biomechanics of the horse’s back helps to understand how it works and to ensure its physical integrity [3]. Horses’ spinal column is composed of seven cervical, eighteen thoracic, six lumbar, five sacral and fifteen to twenty-one coccygeal vertebrae. The area of the horse’s back corresponding to the thoracolumbar region is the most mobile, particularly between the fourteenth thoracic vertebra (T14) and the eighteenth thoracic vertebra (T18) [4]. The very large volume and weight of a horse’s visceral mass exert high stresses on the spinal column due to the inertia of these moving fluid masses during locomotion [3]. 

The main movements visible on a horse’s back are movements of flexion (ventral bending with a dorsal convexity) and extension (dorsal bending with a ventral convexity), occurring in the median plane [1]. During walking and trotting, the thoracolumbar spine undergoes two cycles of flexion/extension per stride, and during galloping, the spine undergoes one movement of flexion/extension per stride [5].

Back lesions in equine athletes are a frequent cause of reduced performance [6,7]. Studies showed that the most common horseback lesions are dorsal spinous processes impingement and osteoarthritis of intervertebral synovial joints; however, soft tissue lesions, such as supraspinous ligament damage or muscle strains, can also be observed [8,9,10]. These lesions cause pain and abnormal reactions in horses. Horses are considered pathologic if they show clear signs of pain/discomfort on palpation and when these reactions do not decrease with repeated palpation [1]. Pathological horses modify their neuromuscular control of walking and trotting to cope with back pain and decrease their back’s range of motion. Due to back pain, flexion/extension movements are reduced to limit the displacement of the individual segments of the back. The reduction in the range of back flexion/extension involves a decrease in stride length and the amplitude of protraction and retraction movements [1]. For all these reasons, evaluating horses’ back mobility is a major concern for the improvement of animal welfare.

Currently, back mobility is visually assessed by veterinarians during a locomotor examination. Back mobility grades are attributed to different conditions [11], but agreements in the visual scoring of equine back mobility are uncommon and the gradation methods remain subjective [12]. This leads to the need for the development of a method to repeatedly quantify equine back motion.

A previous study measured the flexion and extension range of motion directly from fresh carcasses (ex vivo) [4]. On each specimen, the flexion and extension movements of the spine were reproduced using elastic straps attached to anatomical insertions of the trunk muscles. Forces were applied to the straps to induce these movements. These straps were stretched between the sternum and pubis in order to reproduce the flexion movement induced by the rectus abdominis muscle. Other straps were stretched between the *tuber sacrale* and the first three thoracic spinous processes to reproduce the extension movement of the erector spinae muscle. The authors considered a neutral position of the trunk (free and unrestrained) as a zero. They derived the maximum angle of flexion and the maximum angle of extension, combining both to determine the total range of flexion/extension. The measurements of intervertebral mobility showed that the mean range of flexion was 12.3° in the lumbosacral joint and 3.6° in the thoracolumbar region (between T17 and T18 and between T18 and L1). The segmental intervertebral range of extension varied from 4° to 14° in the lumbosacral joint and varied from 0.3° to 1.8° in the thoracic region (between T14 and T18), according to the applied stress [4].

Other studies measured the flexion and extension ranges of motion in vivo using a motion capture system (MOCAP) [1,5,13,14,15]. These studies showed flexion/extension ranges of motion of approximately 4° between T6, T13, and T18 while trotting in a straight line on a hard surface [1]; 4.91° and 4.97° between the withers, T15 and the pelvis, while trotting in a straight line on hard and soft surfaces, respectively [5]; 3.07° at T17 (rotation around the mediolateral axis of the T17 markers) during trotting in a straight line on a hard surface [13]; and 2.9° between T13, T18, and the pelvis during trotting in a straight line on a hard or soft surface [14]. Recently, a study showed flexion/extension ranges of motion of 3.86° and 4.05° during walking, 5.96° and 5.66° during trotting, and 9.15° and 9.17 during galloping between the withers, T15, and the pelvis in a circle, respectively, at the left and right rein on a soft surface [15].

MOCAP allows the monitoring of these spatiotemporal events [16], including thoracolumbar angle evolution [5]. Although these systems are highly reliable, they require laboratory conditions with exercises performed with a limited volume. These conditions do not reflect the full spectrum of exercises needed by the clinician during a complete locomotor examination. The back movements of the equine athlete during sports and racing activities are also different from these laboratory conditions.

Many studies have demonstrated that inertial measurement units (IMUs) are a real alternative to motion capture systems [17]. The use of IMUs allows the recording of the horse’s locomotion with onboard technologies and in field conditions [18,19,20,21,22,23,24,25,26]. Stance-phase detection methods from IMUs mounted on canon bones [18,21,22], pasterns [22], and hooves [22,23,24] have been recently developed. IMUs also allow the measuring of protraction and retraction angles [19]. Some studies also developed asymmetry quantification methods to assist with veterinarian examinations [20,25,26].

Martin et al. (2014) [27] compared a back flexion/extension quantification method from IMUs and from MOCAP. The method was developed using a single horse during walking, trotting, and galloping on a treadmill. The authors calculated the relative orientation between two IMUs mounted on the T12, T16, and L2 vertebrae (the angle formed between T12 and 16 and between T16 and L2 vertebrae). A calculation based on the orientational difference of both IMUs allowed for obtaining a thoracic and a thoracolumbar angle. During trotting, the accuracy (mean bias ±SD) between the IMUs and MOCAP methods were, respectively, for the thoracic and thoracolumbar angles, 0.57° ± 0.44° and 0.65° ± 0.47°.

Another study used IMUs to assess the back movement of horses, but it focused on vertical displacements and the flexion and extension angles were not assessed [28]. Recently, a study reported the relative change in orientation between pairs of skin-mounted IMUs, between T13 and T18, for example [29]. This study looked for local angles at a specific point, while our study tried to determine the global mobility of the back to reproduce what veterinarians subjectively evaluate.

However, no method dedicated to quantifying a global angle of flexion and extension, reflecting global back mobility in field conditions has been addressed yet.

It would be very useful if this type of measurement could be carried out using the locomotor asymmetry quantification systems that already use two IMUs on the withers and pelvis. In the present study, a third IMU was positioned on the T18 vertebra in order to evaluate back mobility in relation to the extremities of the trunk (withers and pelvis).

In this context, the aim of this study was to evaluate a new method of back flexion/extension quantification from inertial measurement units mounted on a horse’s withers, thoracolumbar region, and *tuber sacrale* of the pelvis, compared to motion capture (MOCAP) used as a gold standard.

## 2. Materials and Methods

### 2.1. Horses

Four sound horses (one thoroughbred gelding, one thoroughbred mare, one French saddle mare, and one French trotter mare (height 162 ± 5 cm (mean ± SD), age 10 ± 3 years (mean ± SD)) were included in the study. All horses used in this study were familiar with galloping, regularly ridden, and trained at the three gaits. Prior to the procedure, the protocol was examined and approved by the dedicated clinical research ethics committee (Comité d’Ethique en Recherche Clinique, ENVA, N° 2022-09-19).

### 2.2. Data Acquisition

**Figure 1:**
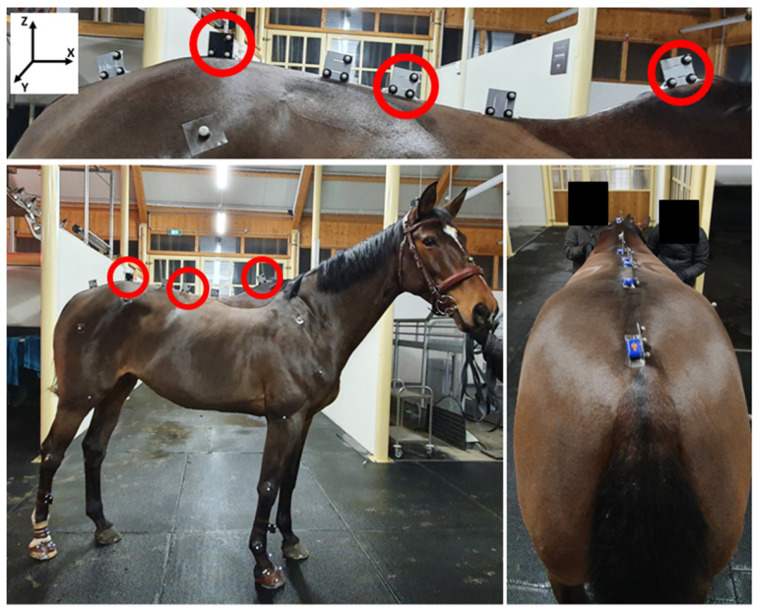
IMU and marker localization on the horse.

Horses were equipped with reflective markers and IMUs (Figure 1).

Three synchronized IMUs (Blue Trident, dual-g sensors, Vicon Motion Systems Ltd., Oxford, UK) with a full-scale range of 16 g (Low-g), 2000°/s, 16 bits, sampling at 225 Hz, were used. IMUs were positioned on the withers, thoracolumbar region (T18 vertebra), and the *tuber sacrale* of the pelvis of the horse (Figure 1, circles). IMUs were placed via manual palpation by the same expert veterinarian for each horse. The other IMUs, observable in Figure 1, were part of a larger study and were not used in the present study. Only one additional IMU (positioned on the right forelimb pastern) was only used for synchronizing both systems.

**Figure 2:**
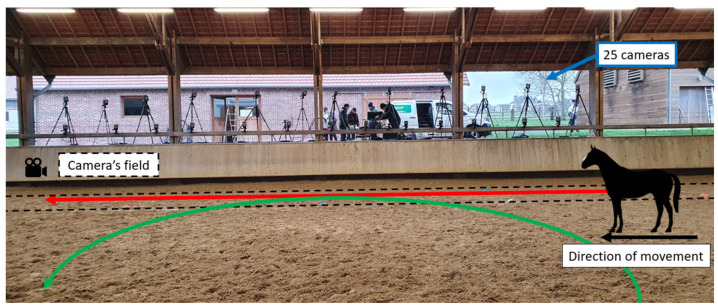
Positioning of the Vicon cameras on one side of the indoor arena. The black dotted lines correspond to the camera’s field. The trajectory of the straight line during walking and trotting is represented by the red arrow, and the trajectory of the circle during galloping is represented by the green arrow.

For the motion capture system, twenty-five synchronized cameras (Vicon, Oxford Metrics Ltd., Oxford, UK), sampling at 200 Hz, were set up on one side of the indoor arena (Figure 2). For each IMU used in this study, three kinematics markers were glued to the side of the IMU using an aluminum plate (Figure 1, circles). The most ventral and cranial marker was used to locate the position of the IMU. One additional free marker was used to synchronize the IMUs with MOCAP. To synchronize both systems, an additional reflective marker was used to strike the IMU positioned on the pastern at the beginning and at the end of each trial. The other markers, observable in Figure 1, were not used in this study.

Only the right side of the horses was equipped. The horse’s equipment and the camera’s positioning choices were performed to prioritize the length of the field. Approximately, the camera’s field of capture was 18 m.

Each horse performed walking and trotting passages in hand, moving in straight lines. The galloping passages were performed while lunging in a large left rein circle, approximately twenty meters in diameter. While lunging, the handler steered the horse towards the camera’s field for a few strides tangential to the rectangular measurement field. Only the full strides performed into the camera field were considered. All the experimentation took place in a covered area on a soft surface (sand). For the left circles, the IMU and markers (attached to the right side of the horse) were positioned outside the circle to be captured by the cameras.

### 2.3. Data Processing

Following the data acquisition, the data processing included two steps, each corresponding to the two types of raw data: the motion capture data and the IMUs data. Except for the preliminary step of labelling the motion capture data, all the motion capture and IMU data were processed using calculation software (Matlab R2021b, The MathWorks Inc., Natick, MA, USA).

#### 2.3.1. Motion Capture Data Processing

First, the raw motion capture data needed the preliminary step of labelling. With the help of the manufacturer’s dedicated software (Nexus 2.14.0, Oxford Metrics Ltd., Oxford, UK), the 3D coordinates for each reflective marker of interest were recovered.

Then, the angle of flexion/extension between the withers, thoracolumbar (T18), and pelvis markers was calculated. A fourth-order Butterworth low-pass filter with a 5 Hz cut-off frequency was applied to the 3D data of the withers, thoracolumbar, and pelvis markers. From the filtered data, the angle of flexion/extension was obtained using vectors (Equations (1) and (2)), vectors norms (Equations (3) and (4)), and scalar products (Equation (5)). The final angle (AngleMocap) was obtained from the (Equation (6)).

(1) $$\vec{V_{Withers}}\;\;\left(\begin{array}{c} x\\ y\\ z \end{array}\right)=\left(\begin{array}{c} x_{T18}-x_{Withers}\\ y_{T18}-y_{Withers}\\ z_{T18}-z_{Withers} \end{array}\right)$$

(2) $$\vec{V_{Pelvis}}\;\;\left(\begin{array}{c} x\\ y\\ z \end{array}\right)=\left(\begin{array}{c} x_{T18}-x_{Pelvis}\\ y_{T18}-y_{Pelvis}\\ z_{T18}-z_{Pelvis} \end{array}\right)$$

(3) $$the\;\left|\left|\;\vec{V_{Withers}}\;\right|\right|\;\left(\begin{array}{c} x\\ y\\ z \end{array}\right)=\sqrt{\vec{V_{Withers}}{\left(x\right)}^{2}+\vec{V_{Withers}}{\left(y\right)}^{2}+\vec{V_{Withers}}{\left(z\right)}^{2}}$$

(4) $$\left|\left|\;\vec{V_{Pelvis}}\;\right|\right|\;\left(\begin{array}{c} x\\ y\\ z \end{array}\right)=\sqrt{\vec{V_{Pelvis}}{\left(x\right)}^{2}+\vec{V_{Pelvis}}{\left(y\right)}^{2}+\vec{V_{Pelvis}}{\left(z\right)}^{2}}$$

(5) $$\vec{V_{Withers}}\;.\;\vec{V_{Pelvis}}=\vec{V_{Withers}}\left(x\right)\times \vec{V_{Pelvis}}\left(x\right)+\vec{V_{Withers}}\left(y\right)\times \vec{V_{Pelvis}}\left(y\right)+\vec{V_{Withers}}\left(z\right)\times \vec{V_{Pelvis}}\left(z\right)$$

(6) $$\mathrm{AngleMocap}=\;\cos^{-1}\left(\;\frac{\vec{V_{Withers}}\;.\;\vec{V_{Pelvis}}}{||\vec{V_{Withers}}||\times ||\vec{V_{Withers}}||}\right)$$

#### 2.3.2. IMU Data Processing

The tri-axial accelerations were obtained from the raw IMU data in each IMU reference frame. The IMU acceleration data were corrected from a motionless frame (a standing square), using a rotation matrix in order to align the Z axis with the direction of the gravity vector (Equation (7)). We assumed that the rotation of each IMU reference frame around its own Z axis was zero at standing square. Then, the orientation correction angles α and β (regarding the terrestrial X-axis and floating Y-axis, respectively) were obtained for each IMU by solving the system (Equation (8)). Thus, assuming that the norm of the acceleration vector is equal to 1 g motionless, the acceleration values of each IMU at each instant in the same terrestrial reference could be found by multiplying the rotation matrix (with its associated angles α and β) by the raw acceleration values (Equation (9)).

(7) $$\left[\begin{array}{c} 0\\ 0\\ -g \end{array}\right]=\left[\begin{array}{ccc} \cos\left(\beta \right) & 0 & \sin\left(\beta \right)\\ \sin\left(\alpha \right)\times \sin\left(\beta \right) & \cos\left(\alpha \right) & -\sin\left(\alpha \right)\times \cos\left(\beta \right)\\ -\cos\left(\alpha \right)\times \sin\left(\beta \right) & \sin\left(\alpha \right) & \cos\left(\alpha \right)\times \cos\left(\beta \right) \end{array}\right]*\left[\begin{array}{c} \gamma_{x}\\ \gamma_{y}\\ \gamma_{z} \end{array}\right]$$

with *γ_x_, γ_y_ et γ_x_* the 3D acceleration signals of the IMU at a motionless instant in the IMU reference frame

(8) $$\left\{\begin{array}{c} \cos\left(\beta \right)\times \gamma_{x}+\sin\left(\beta \right)\times \gamma_{z}=0\\ \sin\left(\alpha \right)\times \sin\left(\beta \right)\times \gamma_{x}+\cos\left(\alpha \right)\times \gamma_{y}-\sin\left(\alpha \right)\times \cos\left(\beta \right)\times \gamma_{z}=0\\ \;-\cos\left(\alpha \right)\times \sin\left(\beta \right)\times \gamma_{x}+\sin\left(\alpha \right)\times \gamma_{y}+\cos\left(\alpha \right)\times \cos\left(\beta \right)\times \gamma_{z}=-g \end{array}\right.\;\mathrm{Giving}:\;\beta =\tan^{-1}\left(\;\frac{-\gamma_{x}}{\gamma_{z}}\;\right)\;\mathrm{and}\;\alpha =\tan^{-1}\left(-\gamma_{y}.\left(\frac{1}{\left(\sin\left(\beta \right).\gamma_{x}\right)-\left(\cos\left(\beta \right).\gamma_{z}\right)}\right)\;\right)$$

(9) $$\left[\begin{array}{c} \gamma_{x}\;R_{0}\\ \gamma_{y}\;R_{0}\\ \gamma_{z}\;R_{0} \end{array}\right]=\left[\begin{array}{ccc} \cos\left(\beta \right) & 0 & \sin\left(\beta \right)\\ \sin\left(\alpha \right)\times \sin\left(\beta \right) & \cos\left(\alpha \right) & -\sin\left(\alpha \right)\times \cos\left(\beta \right)\\ -\cos\left(\alpha \right)\times \sin\left(\beta \right) & \sin\left(\alpha \right) & \cos\left(\alpha \right)\times \cos\left(\beta \right) \end{array}\right]*\left[\begin{array}{c} \gamma_{x}\;R_{IMU}\\ \gamma_{y}\;R_{IMU}\\ \gamma_{z}\;R_{IMU} \end{array}\right]$$

With *γ_x_ R_IMU_, γ_y_ R_IMU_ et γ_x_ R_IMU,_* the 3D acceleration signals of the IMU in the IMU reference frame.

And *γ_x_ R_0_, γ_y_ R_0_ et γ_x_ R_0_* the 3D acceleration signals of the IMU in the terrestrial reference frame.

In the following steps, only the IMU data corrected in the terrestrial reference frame were used. The next step of IMU data processing consisted of obtaining the vertical displacement of the withers, the thoracolumbar, and the pelvis IMUs. For this, the acceleration signal measured was integrated twice and high-pass filtered using a fourth-order Butterworth filter with a cutoff frequency set to 1 Hz to obtain the displacement curves, as described in Macaire et al. (2022) [20].

**Figure 3:**
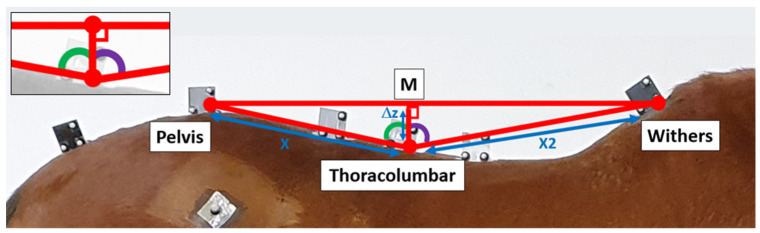
Scheme of the back flexion/extension quantification method from the IMUs.

The last step of the calculation used trigonometry to obtain the flexion/extension angle at the level of the thoracolumbar region. The scheme of the method can be found in Figure 3. Computing the mean value between the vertical displacement of the withers and the pelvis, the vertical displacement of the M point was obtained at each instant of time. The Δz distance was then obtained by subtracting the vertical displacement of the M point and the vertical displacement of the thoracolumbar point at the same instant of time. The X and X2 distances were measured by hand using a tape measure on the stationary horse.

The angles of “pelvis/T18/M” and “withers/T18/M”, depicted as the green and purple angles in Figure 3, were calculated using Equations (10) and (11). The final angle (AngleIMU) was calculated by adding the green and purple angles (Equation (12)).

(10) $${\mathrm{Angle}}_{\mathrm{Withers}\_T18\_M}=\cos^{-1}\left(\frac{\Delta z}{X2}\;\right)$$

(11) $${\mathrm{Angle}}_{\mathrm{Pelvis}\_T18\_M}=\cos^{-1}\left(\frac{\Delta z}{X}\;\right)$$

(12) $$\mathrm{AngleIMU}={\mathrm{Angle}}_{\mathrm{Withers}\_T18\_M}+{\;\mathrm{Angle}}_{\mathrm{Pelvis}\_T18\_M}$$

Finally, a fourth-order Butterworth filter with a 5 Hz cut-off frequency was applied to the obtained final IMU angle corresponding to the flexion/extension of the back.

#### 2.3.3. Synchronization and Homogenization of Data Sets

Both data sets were homogenized at 200 Hz using a continuous derivative interpolation function to obtain only one standardized sampling frequency. Then, the two data sets were synchronized using the strike of synchronization at the beginning and the end of each trial with one reflective marker on the pastern. The data were cut down to keep only the data between these two peaks on each data set.

#### 2.3.4. Determination of the Range of Flexion and Extension

**Figure 4:**
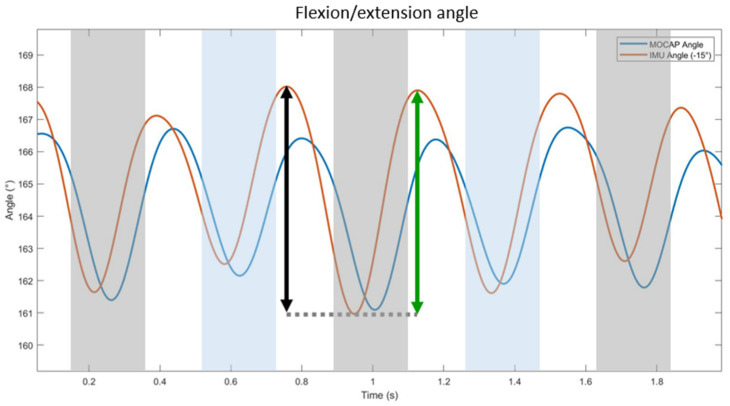
Illustration of the back flexion/extension angles calculated using MOCAP (blue curve) and IMUs (orange curve) on one horse (Horse n° 1) trotting in a straight line. The black arrow indicates the amplitude of extension, and the green arrow indicates the amplitude of flexion. The gray areas represent the left forelimb stance phase, and the blue areas represent the right forelimb stance phase.

The angles obtained using motion capture (“AngleMocap”) and IMUs (“AngleIMU”) follow sinusoidal patterns, from which a range of flexion and extension of the back can be calculated (Figure 4).

The range of flexion is the difference between the maximal and minimal value of the upward motion during the propulsion phase (Equation (13)). The range of extension is the difference between the maximal and minimal value of the downward motion during the damping phase (Equation (14)).

(13) $$Range_{Flexion}\;\left(i\right)=max\;\left(Angle\right)\left(i\right)-min\;\left(Angle\right)\left(i\right)$$

(14) $$Range_{Extension}\;\left(i\right)=max\;\left(Angle\right)\left(i-1\right)-min\;\left(Angle\right)\left(i\right)$$

### 2.4. Statistical Analysis

The ranges of flexion and extension obtained using the IMU method were compared with the ranges of flexion and extension obtained using method. First, the Bland-Altman’s method was used for comparison [30,31,32]; then, the constant and proportional biases were determined from an ordinary least product regression [33,34,35,36,37], and finally, Pearson’s linear correlation coefficient [38,39] was calculated.

#### 2.4.1. Bland-Altman’s Method

The agreement among the two measurement techniques (the developed method with the IMU and the reference method with motion capture) was evaluated using Bland-Altman’s method [30,31,32], meaning the precision and accuracy of the range of flexion and extension were studied. With this method, each of the n samples is represented on a graph by assigning the mean of the two measurements as the x-value and the difference between the two measurements as the y-value.

These equations describe the Bland-Altman’s method:

(15) Difference=Developed Method−Reference Method

(16) $$Bias=mean\;\left(Difference\right)$$

(17) $$SD=standard\;deviation\;\left(Difference\right)$$

(18) LimitAgreementHigh=Bias+1.96×SD

(19) LimitAgreementLow=Bias−1.96×SD

The accuracy, i.e., bias (Equation (16)), corresponded to the mean difference between the developed method values and reference method values. The precision corresponded to the standard deviation of the differences (SD) (Equation (17)). The limits of agreement (Equations (18) and (19)) were also calculated, defined as the range including 95% of the difference values. All the results were expressed in degrees.

#### 2.4.2. Ordinary Least Product Regression

To determine the constant and proportional bias, the ordinary least product regression method was used [33,34,35,36,37]. The biases were calculated using the Matlab software functions “fitlm” [40] and “coefCI” [41] (Matlab R2021b, The MathWorks Inc., Natick, MA, USA). The degree of constant bias was calculated using the 95% confidence interval (95% CI) of the y-intercept. If the 95% CI for the intercept included the value of zero, there was no constant bias. The proportional bias was determined from the 95% CI of the slope. If the 95% CI of the slope included the value of 1.0, there was no proportional bias.

#### 2.4.3. Correlation

To study the link between both methods, linear regression curves [38] were plotted. Pearson’s correlation coefficient (Pearson’s *r*) [39] was calculated using the Matlab software function “corr” (Matlab R2021b, The MathWorks Inc., Natick, MA, USA). Pearson’s correlation coefficient was compared to the thresholds defined by Cohen (1992) to evaluate the relationship’s strength (0–0.1: none; 0.1–0.3: small; 0.3–0.5: medium; >0.5: large) [42].

## 3. Results

The number of flexion/extension movements captured using both systems is shown in Table 1. The analyzed strides were selected when the gait was stabilized. The number of strides per horse and per gait could be random, depending on the number of full strides performed into the camera field. This information was not available prior to the start of the processing of the data.

The mean range of flexion and the mean range of extension for each gait from both methods are shown in Table 2.

**Figure 5:**
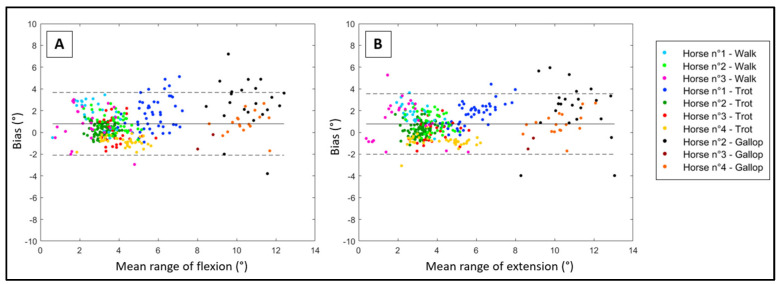
Bland–Altman’s plot (in degrees (°)) of the reference (MOCAP) range of flexion (**A**) and extension (**B**) and the range of flexion and extension obtained using the developed method (IMU). One color represents one horse at one gait.

**Figure 6:**
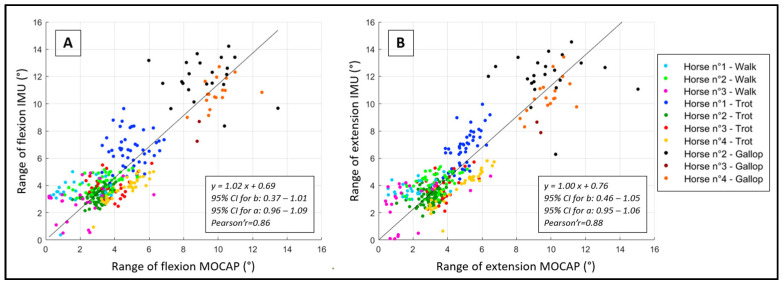
Pearson’s correlation curves between the reference (MOCAP) range of flexion (**A**) and extension (**B**) and the range of flexion and extension obtained using the developed method (IMU). One color represents one horse at one gait.

For the range of flexion, the precision and accuracy of the developed method are shown in Table 3 and Figure 5A. Whatever the condition, the average bias was less than 0.8° (±1.5°). There was a constant bias of 0.69° (95% CI 0.37 to 1.01) between the developed method and the reference method. However, there was no proportional bias (bias of 1.02°, 95% CI 0.96 to 1.09). The correlation curve between both methods is shown in Figure 6A, with a large Pearson’s *r* of 0.86.

For the range of extension, the precision and accuracy of the developed method are shown in Table 3 and Figure 5B. Whatever the condition, the average bias was less than 0.8° (±1.4°). There was a constant bias of 0.76° (95% CI 0.46 to 1.05) between the developed method and the reference method. However, there was no proportional bias (bias of 1.00°, 95% CI 0.95 to 1.06). The correlation curve between both methods is shown in Figure 6B, with a large Pearson’s *r* of 0.88.

## 4. Discussion

In this study, an innovative method of back flexion/extension quantification was developed based on only three IMUs located on the withers, the thoracolumbar region, and the *tuber sacrale* of the pelvis for walking, trotting, and galloping in field conditions. Only three IMUs were used because we aimed to develop a simple and functional tool that provides precise movement quantification. To do that, we needed to verify that the range of flexion/extension measured with three IMUs matches the measurements collected with a MOCAP gold standard method and with previously published data.

Denoix (1999) found a total range of flexion/extension of 26.3° in the lumbosacral joint and a total range of flexion/extension of 5.4° in the thoracolumbar region (between the T17 and T18 and between the T18 and L1 vertebrae) from fresh carcasses [4]. Our method measures a global back angle between the withers, thoracolumbar region, and pelvis, which is not directly comparable with the range of motion found by Denoix (1999). In our study, the maximum range of motion measured with both systems was 9.8° (±1.4°) using the MOCAP system and 11.4° (±1.7°) using the IMUs during galloping. Although Denoix (1999) measured an intervertebral angle whereas we measured a global back angle, our results appear to be consistent with studies carried out on isolated anatomical parts.

A comparison of our results with other in vivo kinematic studies shows that the amplitude of flexion/extension measured in our study in the thoracolumbar region is also consistent with previously published work. During trotting, our results using MOCAP showed a mean range of flexion of 3.9° (±1.1°) and a mean range of extension of 3.9° (±1.1°). Compared to other studies that found mean ranges of flexion/extension between 2.9° [14] and 5.0° [5] during trotting in a straight line, our results are in the same range. During galloping, our results showed a mean range of flexion of 9.5° (±1.3°) and a mean range of extension of 9.8° (±1.4°) in a large circle in the left rein. These results are consistent with the results of Egenvall et al. (2022) [15], who found a mean range of flexion/extension of 9.2° in the same conditions [15]. These converging results with MOCAP allow us to compare our IMU results to the MOCAP results, which could be considered a reliable reference.

Our developed method using IMUs has the advantage of being applicable in field conditions in comparison to our MOCAP method, which requires laboratory conditions. The IMU method showed a mean bias of 0.8° (±1.5°) for flexion movements and a mean bias of 0.8° (±1.4°) for extension movements, all gaits combined, in comparison with the MOCAP method using Bland–Altman’s comparison. In a previous study [27], the mean bias of the thoracolumbar angles between the IMU and MOCAP methods was 0.65° (±0.47°) during trotting and 0.63° (±0.44°) during galloping. In this study, the data were recorded only using one horse on a treadmill involving a stabilized gait and more repeatable strides between themselves. In our study, four horses were practiced in real conditions involving variable speeds and strides.

The range of flexion and range of extension were calculated separately in our study. We noticed a mean difference between the range of flexion and extension of 0.3° from MOCAP and 0.1° from the IMUs. Nevertheless, the bias measured between MOCAP and IMUs remains the same, with 0.8° of bias for both movements.

The ordinary least product regression method showed no proportional bias between both methods and a constant bias of 0.69° and 0.76° for the range of flexion and extension, respectively. These results demonstrate the existence of a linear relationship between both methods. The range of flexion and extension calculated with the developed method (IMUs) was consistently slightly superior to the range of flexion and extension obtained using the reference method (MOCAP). This constant bias was small (less than 1°) but cannot be completely ignored, given the small amplitude of flexion-extension movements of the back. In a clinical context, however, the essential result is the absence of proportional bias demonstrated in this study, given that the crucial point is to be able to measure the variation of movements in different circumstances or during longitudinal monitoring. Absolute bias should not be a major problem as long as it is constant.

This study presents some limitations. First, only four sound horses were included in the study. This small amount of collected data does not present significant population diversity. However, analyzing the variability of back mobility in a large population of horses was not the purpose of the study. In this study, the first objective was to validate a method with a sufficient number of back movements. We were able to analyze 340 movements of flexion/extension, all gaits confused, and all horses combined.

Another limitation of our study is the prerequisites to use this method. Indeed, we need to know the distance between the IMU placed on the withers, the thoracolumbar region, and the distance between the IMU placed on the thoracolumbar region and the pelvis. It is also important that the horses stand still for a short moment to be able to correct the orientation of the sensors using the gravity acceleration measurement. These are two essential prerequisites for the quality of the measurements.

For the galloping conditions, only the left rein circle was tested in this study. The gallop is an asymmetric gait with a different mechanism between the left and right rein. In this study, horses galloped in very wide circles with recording zones tangential to the straight lines. In these conditions, we assumed that the range of flexion/extension would be similar between the two reins for sound horses. Applying this method to tighter curves would require further validation in order to ensure that the inclination of the horse and the back curvature (lateroflexion) within the curves did not influence the IMU results.

## 5. Conclusions

To conclude, this study presents an original method to quantify horses’ back mobility during walking, trotting, and galloping in veterinarian examination conditions (walking and trotting in a straight line and galloping in a circle). The use of IMUs to quantify horses’ back mobility would help veterinarians in their diagnostics. It would also help them to measure the evolution of horses’ back mobility in the case of the longitudinal monitoring of the horse. The range of flexion/extension measured using MOCAP and IMUs reflects the range of flexion/extension measured in other studies and reflects anatomical reality.

The next step of this study would be to calibrate the measurement by comparing it with a clinical opinion, which should render it possible to discriminate between sound and pathological horses. Thus, in future plans, the method developed using IMUs could be compared with the grade of dorsal restriction visually attributed by veterinarians during the examination of horses suffering from back pain. The correlations with the subjective observations by specialist veterinarians could provide us with information on the robustness and reliability of our method for detecting restricted back mobility. Finally, this method could be used in a rehabilitation protocol of dorsalgic horses to realize a longitudinal evaluation of their back mobility.

## Figures and Tables

**Table 1:** Number of flexion/extension movements analyzed in the presented data set.

Gait	Horse	Number of Flexion/Extension Movements
Walk	Horse 1	26
	Horse 2	42
	Horse 3	24
Trot	Horse 1	48
	Horse 2	88
	Horse 3	26
	Horse 4	38
Gallop	Horse 2	26
	Horse 3	2
	Horse 4	20

**Table 2:** Comparison of the mean (±SD) range of flexion/extension of the back obtained using motion capture and IMUs (in degrees (°)).

Gait	Motion Capture		IMUs	
	Flexion	Extension	Flexion	Extension
Walk	2.6° (±1.3°)	2.5° (±1.3°)	3.7° (±1.1°)	3.8° (±1.1°)
Trot	3.9° (±1.1°)	3.9° (±1.1°)	4.3° (±1.6°)	4.2° (±1.7°)
Gallop	9.5° (±1.3°)	9.8° (±1.4°)	11.3° (±1.6°)	11.4° (±1.7°)

**Table 3:** Bland–Altman’s comparison (in degrees (°)) of the reference range of flexion and extension and the range of flexion and extension obtained using the developed method. The accuracy (bias), precision (SD), upper/lower limits of agreement (ULA and LLA), and Pearson’s correlation coefficient (Pearson’s *r*) are indicated for the range of flexion and extension of the back.

	Bias	SD	ULA	LLA	Pearson’s *r*
Flexion	0.8°	1.5°	3.7°	−2.1°	0.86
Extension	0.8°	1.4°	3.6°	−2.0°	0.88

## Data Availability

Any additional requests can be addressed to the corresponding author.
